# Supplementary material for: COZOID: contact zone identifier for visual analysis of protein-protein interactions
Source: BMC Bioinformatics. 2018 Apr 6;19:125. doi: 10.1186/s12859-018-2113-6 (PMC5889581; doi:10.1186/s12859-018-2113-6)
Supplement: Supplementary file 2 — Software build. Executable binary file of the software tool. (ZIP 118,784 kb) [file 12859_2018_2113_MOESM2_ESM.zip › cozoid/java/docs/beansbinding-1.2.1-doc.zip!/org/jdesktop/el/impl/parser/ELParserTokenManager.html]

ELParserTokenManager (Beans Binding)


---


|  |  |  |
| --- | --- | --- |
| **Overview** \| **Package** \| **Class** \| **Use** \| **Tree** \| **Deprecated** \| **Index** \| **Help** | |  |
| **PREV CLASS**   **NEXT CLASS** | **FRAMES**    **NO FRAMES**     **All Classes** |  |
| SUMMARY: NESTED \| FIELD \| CONSTR \| METHOD | DETAIL: FIELD \| CONSTR \| METHOD |  |


---


## org.jdesktop.el.impl.parser Class ELParserTokenManager

```
java.lang.Object
  org.jdesktop.el.impl.parser.ELParserTokenManager
```

**All Implemented Interfaces:**: ELParserConstants

---

``` public class ELParserTokenManager extends java.lang.Object implements ELParserConstants ```

---

| **Field Summary** | |
| --- | --- |
| `protected  char` | `curChar` |
| `java.io.PrintStream` | `debugStream` |
| `protected  SimpleCharStream` | `input_stream` |
| `static int[]` | `jjnewLexState` |
| `static java.lang.String[]` | `jjstrLiteralImages` |
| `static java.lang.String[]` | `lexStateNames` |

**Fields inherited from interface org.jdesktop.el.impl.parser.ELParserConstants**

`AND0, AND1, BADLY_ESCAPED_STRING_LITERAL, COLON, COMMA, DEFAULT, DIGIT, DIV0, DIV1, DOT, EMPTY, END_EXPRESSION, EOF, EQ0, EQ1, EXPONENT, FALSE, FLOATING_POINT_LITERAL, FUNCTIONSUFFIX, GE0, GE1, GT0, GT1, IDENTIFIER, ILLEGAL_CHARACTER, IMPL_OBJ_START, IN_EXPRESSION, INSTANCEOF, INTEGER_LITERAL, LBRACK, LE0, LE1, LETTER, LITERAL_EXPRESSION, LPAREN, LT0, LT1, MINUS, MOD0, MOD1, MULT, NE0, NE1, NOT0, NOT1, NULL, OR0, OR1, PLUS, QUESTIONMARK, RBRACK, RPAREN, START_DEFERRED_EXPRESSION, START_DYNAMIC_EXPRESSION, STRING_LITERAL, tokenImage, TRUE`


**Constructor Summary**

`ELParserTokenManager(SimpleCharStream stream)`

`ELParserTokenManager(SimpleCharStream stream,
int lexState)`


| **Method Summary** | |
| --- | --- |
| `Token` | `getNextToken()` |
| `protected  Token` | `jjFillToken()` |
| `void` | `ReInit(SimpleCharStream stream)` |
| `void` | `ReInit(SimpleCharStream stream, int lexState)` |
| `void` | `setDebugStream(java.io.PrintStream ds)` |
| `void` | `SwitchTo(int lexState)` |

**Methods inherited from class java.lang.Object**

`clone, equals, finalize, getClass, hashCode, notify, notifyAll, toString, wait, wait, wait`

**Field Detail**

### debugStream

```
public java.io.PrintStream debugStream
```

---


### jjstrLiteralImages

```
public static final java.lang.String[] jjstrLiteralImages
```

---


### lexStateNames

```
public static final java.lang.String[] lexStateNames
```

---


### jjnewLexState

```
public static final int[] jjnewLexState
```

---


### input\_stream

```
protected SimpleCharStream input_stream
```

---


### curChar

```
protected char curChar
```


**Constructor Detail**

### ELParserTokenManager

```
public ELParserTokenManager(SimpleCharStream stream)
```

---


### ELParserTokenManager

```
public ELParserTokenManager(SimpleCharStream stream,
                            int lexState)
```


**Method Detail**

### setDebugStream

```
public void setDebugStream(java.io.PrintStream ds)
```

---


### ReInit

```
public void ReInit(SimpleCharStream stream)
```

---


### ReInit

```
public void ReInit(SimpleCharStream stream,
                   int lexState)
```

---


### SwitchTo

```
public void SwitchTo(int lexState)
```

---


### jjFillToken

```
protected Token jjFillToken()
```

---


### getNextToken

```
public Token getNextToken()
```


---


|  |  |  |
| --- | --- | --- |
| **Overview** \| **Package** \| **Class** \| **Use** \| **Tree** \| **Deprecated** \| **Index** \| **Help** | |  |
| **PREV CLASS**   **NEXT CLASS** | **FRAMES**    **NO FRAMES**     **All Classes** |  |
| SUMMARY: NESTED \| FIELD \| CONSTR \| METHOD | DETAIL: FIELD \| CONSTR \| METHOD |  |


---
